# Spectrum of Developmental Dental Anomalies in Primary Dentition: A Case Series

**DOI:** 10.7759/cureus.84003

**Published:** 2025-05-13

**Authors:** Mridula Goswami, Archana Singh

**Affiliations:** 1 Pediatric and Preventive Dentistry, Maulana Azad Institute of Dental Sciences, New Delhi, IND

**Keywords:** developmental anomalies, pediatric dentistry, primary teeth, talon cusp, taurodontism

## Abstract

Developmental anomalies in primary teeth present a spectrum of rare conditions, making diagnosis and management challenging for pediatric dentists. This case series explores such anomalies each characterized by unique etiological factors and clinical manifestations. These developmental anomalies, although rare, present significant challenges due to their impact on function, occlusion, and aesthetics in affected children. The uncommon nature of these conditions often makes early diagnosis and effective treatment planning challenging, necessitating specialized knowledge and expertise from pediatric dentists. Etiological factors vary from genetic predispositions to environmental influences, necessitating a comprehensive approach to diagnosis through clinical and radiographic assessments. Treatment strategies aim to preserve tooth structure, restore function, and ensure optimal oral health outcomes. The outcome of this case series emphasizes the critical role of pediatric dentists and general dentists in the management of developmental anomalies in primary dentition. It highlights how an early diagnosis and timely intervention can effectively minimize potential complications and support favorable long-term oral health outcomes in children.

## Introduction

Tooth formation is a highly controlled, complex biological process that depends on a variety of conditions for success. Any disruptions during tooth growth, clinical alterations, or alternations will become apparent. Both genetic and environmental factors contribute to its development [1]. Several genetic mutations have been recognized as contributing factors affecting tooth number and shape by disrupting the molecular signaling pathways involved in early tooth development. Environmental influences, such as the long-term use of antibiotics like tetracycline in young children, excessive fluoride exposure, and inadequate nutrition, also play a role [1]. Anomalies in primary teeth are relatively uncommon. Clinical and radiographic evaluations in multiple epidemiological studies [2-4] have assessed the prevalence of dental abnormalities in children, with reported rates varying globally from 0.012% to 6%. The anomalous teeth are frequently asymptomatic and can be detected during a clinical and radiological examination of the oral cavity [4].

Numerical and morphological developmental dental anomalies in the primary dentition, though relatively uncommon, can significantly influence the developing occlusion and overall oral health of pediatric patients. Numerical anomalies include supernumerary teeth (hyperdontia) and congenitally missing teeth (hypodontia) [5,6]. Supernumerary teeth refer to the presence of extra teeth beyond the normal complement and are reported in approximately 0.4% of the population. These additional teeth can cause crowding, delayed eruption, or displacement of adjacent teeth, potentially complicating the eruption pattern of permanent successors. Conversely, hypodontia, the congenital absence of one or more teeth, affects approximately 0.5% of children and can result in space discrepancies, functional issues, and aesthetic concerns [6]. 

Morphological anomalies such as double teeth and talon cusps also present diagnostic and therapeutic challenges [7]. Double teeth include gemination and fusion. Gemination arises when a single tooth bud attempts to divide, resulting in a tooth with an enlarged crown and typically a single root and root canal. Fusion occurs when two separate tooth germs unite during development, forming a large tooth with a variable root and canal structure. These anomalies appear in approximately 0.4% of the population and may reach up to 2.5% in primary teeth [8]. They can lead to malalignment, delayed exfoliation, or issues in the eruption of the underlying permanent teeth. Another rare morphological anomaly is the talon cusp, observed in about 0.07% of cases [9]. This condition involves the presence of an accessory cusp projecting from the cingulum area to the incisal edge of anterior teeth, often leading to occlusal interference, increased plaque retention, and aesthetic concerns [10]. Overall, the prevalence of developmental anomalies in primary dentition is estimated at 0.54%, compared to 2.5-3.2% in permanent dentition [11]. Although less common, anomalies in primary teeth are of great clinical importance. Timely diagnosis and appropriate intervention are essential, as these early disruptions in tooth development can influence arch integrity, occlusion, and the health of the permanent dentition.

This case series aims to contribute to the existing literature, by the additional evidences, to aid in the identification, diagnosis, and management of developmental dental anomalies within clinical settings. The developmental anomalies are usually associated with the syndrome which is contrary and unique to the present case series as all the mentioned cases are non-syndromic. Understanding these non-syndromic developmental anomalies is particularly important, as they may otherwise be overlooked or misdiagnosed, leading to delayed or inappropriate treatment and future complications.

The case series presents a spectrum of developmental dental anomalies observed in primary teeth, detailing the clinical presentation, diagnostic approaches, and management strategies employed with a comprehensive understanding. This case series will benefit clinicians by contributing to improved long-term oral outcomes in pediatric patients.

## Case presentation

The present case series depicts five cases within the age group of 3-12 years with various developmental anomalies in primary dentition. After obtaining consent, a thorough examination and diagnosis were made. A detailed treatment plan was formulated specific to each case. The treatment was done as per the Declaration of Helsinki protocol. Non-pharmacological management techniques such as communication, Tell-Show-Do, and modeling were implied to achieve cooperation. A thorough medical history, family history, and previous dental history with general physical, clinical, and radiographical evaluation were carried out for each case. A thorough medical history and family history were obtained, and all the cases of developmental anomalies presented in the case series were found to be non-syndromic.

Case 1: gemination and supernumerary tooth

**Figure 1:**
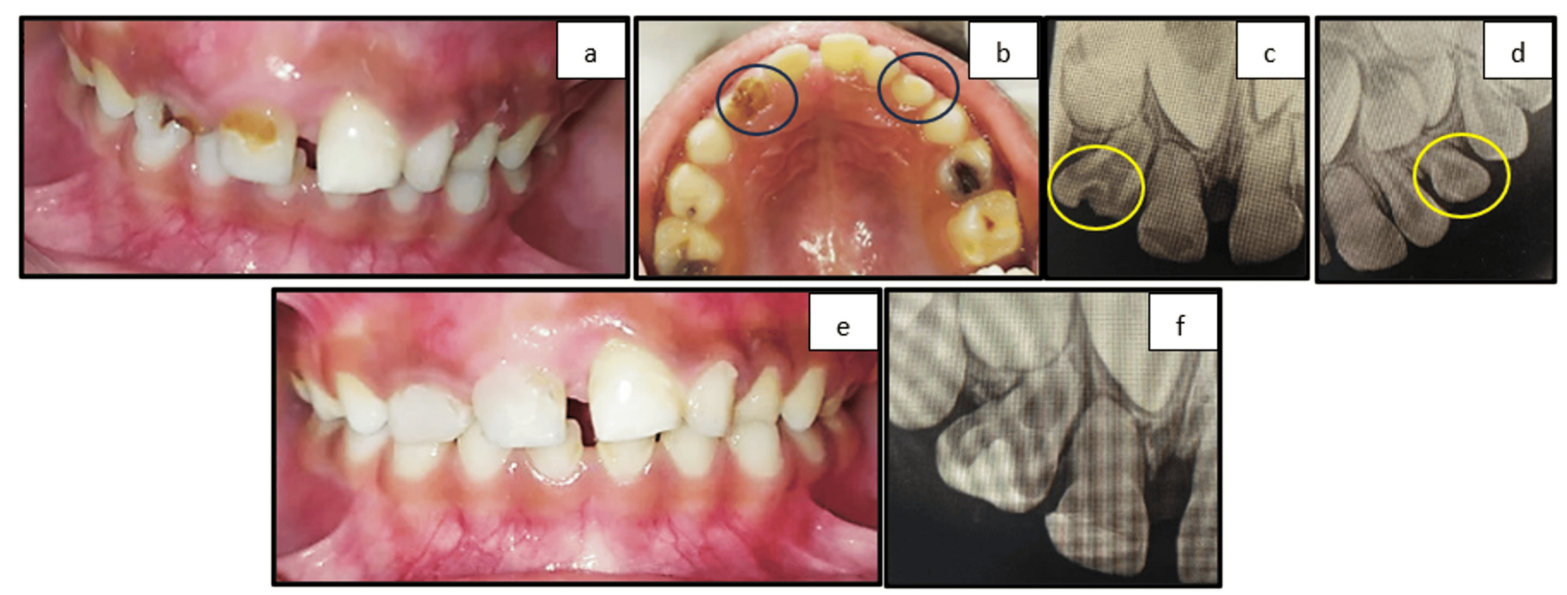
(a) Frontal view showing carious 51 and 52 and gemination of 52. (b) Maxillary occlusal view showing carious 52 and supernumerary tooth between 62 and 63. (c) Radiographic view of gemination with respect to 52. (d) Supernumerary tooth between 62 and 63. (e) Frontal view showing restored 51 and 52. (f) Radiographic view of restored geminated 52

A five-year-old male patient presented with a chief complaint of caries in the maxillary anterior region. Intraoral examination revealed supernumerary teeth present lateral to the left maxillary lateral incisor and carious geminated teeth with respect to the right maxillary lateral incisor (Figure 1a, 1b). A periapical radiograph of the right lateral incisor revealed two crown­-like structures joined together with one common root and pulp canal (Figure 1c). A periapical radiograph of the left maxillary anterior region revealed a supernumerary tooth lateral to the lateral incisor (Figure 1d). The clinical and radiographic findings complemented the diagnosis of gemination of the right lateral incisor and supernumerary teeth in the left anterior region between 62 and 63. The geminated carious teeth were restored with Giomer considering aesthetic concern (Figure 1e, 1f). Other carious teeth were also restored using glass ionomer cement. The left maxillary supernumerary tooth was kept on observation and further follow-up. 

Case 2: fusion of primary and permanent teeth

**Figure 2:**
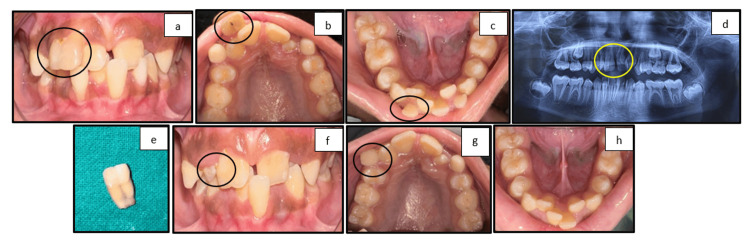
(a) Frontal view showing fused and retained 51 and 52. (b) Maxillary occlusal view showing fused and retained 51 and 52 and erupting 13 and 23. (c) Mandibular occlusal view showing retained 71 and 81. (d) Orthopantomogram showing fused 51 and 52 and fused 11 and 12. (e) Extracted teeth showing fused 51 and 52. (f) Postoperative frontal view showing the crossbite of 21 and 31. (g) Postoperative maxillary view. (h) Postoperative mandibular view showing the healed socket of 31 and 41

A 12-year-old female patient presented with a chief complaint of crowding in the maxillary and mandibular region. On clinical examination, fused 51 and 52 and 71 and 81 were retained along with crowding in both arches. In permanent dentition, crossbite with respect to 11 and 31 and fusion of 11 and 21 teeth were present. An orthopantomogram was done to confirm the clinical diagnosis and to assess the condition of permanent tooth germs. The child was uncooperative in the initial appointment, but cooperative behavior was achieved on further appointments through behavior management techniques such as modeling, Tell-Show-Do, and positive reinforcement. Extractions of fused 51 and 52 and retained 71 and 81 were done after the patch test, and the patient was kept under follow-up (Figure 2).

Case 3: gemination 

**Figure 3:**
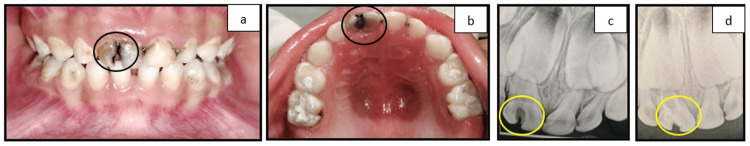
(a) Frontal view showing carious 51 and 52 and gemination of 51. (b) Maxillary occlusal view showing carious 51 and restored 54, 55, 64, and 65. (c) Preoperative radiographic view of geminated 51 with caries involving pulp. (d) Postoperative radiographic view showing obturation in two separate root canals of geminated 51

A five-year-old patient presented with a chief complaint of caries in the maxillary front teeth region for six months. On clinical examination, 51 and 52 were carious and 54, 55, 64, and 65 were restored. The tooth 51 had a larger crown size than the adjacent tooth, and it was diagnosed as gemination after clinical and radiographic evaluation. A periapical radiograph was done with respect to 51 showing caries involving pulp, and it had two separate root canals from the cervical to the apical third of the root. Diagnosis of irreversible pulpitis was made, and a pulpectomy of tooth 51 was done. The patient was clinically and radiographically asymptomatic on further follow-ups (Figure 3).

Case 4: taurodontism

**Figure 4:**
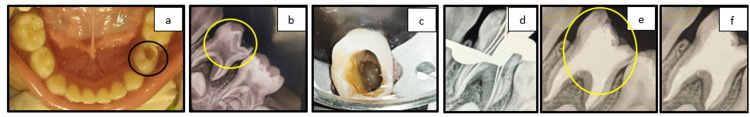
(a) Mandibular occlusal view showing carious 74. (b) Preoperative periapical radiographic view of 74 showing an enlarged pulp chamber with carious pulpal involvement. (c) Access opening under rubber dam showing four root canal orifices of 74. (d) Working length determination periapical radiograph of 74. (e) Periapical radiograph showing an obturated root canal with post-endodontic restoration with respect to 74. (f) One-week follow-up RVG with respect to 74 RVG: radiovisiography

The patient of age four years presented with a chief complaint of acute pain for the last three days with respect to the 74 tooth region. On clinical examination, the tooth was found to be deeply carious approaching pulp. The periapical radiograph evaluation showed dentinal caries involving pulp in 74. On thorough examination, it was found that the pulp chamber of the tooth was apparently larger than the adjacent tooth with tortuous root canals. The tooth was diagnosed as hypotaurodontism (according to Shifman and Chanannel [12]), along with acute irreversible pulpitis. A pulpectomy was done under rubber dam isolation. The negotiation of the tortuous canal was the challenging part of the case which was achieved with proper irrigation using a combination of 1% hypochlorite along with normal saline and careful biomechanical preparation. At one-week follow-up, the patient was clinically and radiographically asymptomatic (Figure 4).

Case 5: talon's cusp (eagle's cusp)

**Figure 5:**
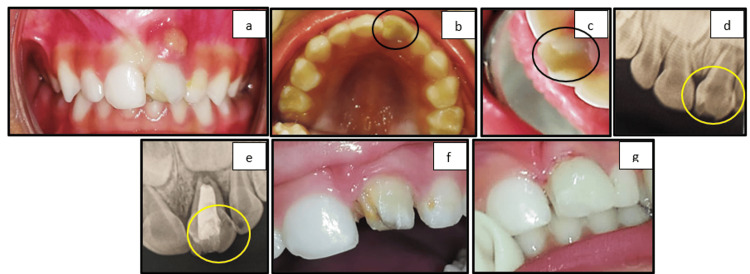
(a) Frontal view showing gingival abscess with respect to 61. (b) Maxillary occlusal view showing talon's cusp with respect to 61. (c) Conical projection over the cingulum region of 61. (d) Preoperative radiograph showing radiolucency in the periapical region of 61. (e) Postoperative radiograph showing the obturated root canal of 61. (f) Crown preparation of 61. (g) Postoperative frontal view showing the strip crown with respect to 61

A three-year-old patient presented with a chief complaint of gingival abscess in the maxillary anterior region for the last two days. On clinical examination, carious 61 with periapical abscess was present. There was the presence of an extra cusp-like projection on the palatal surface of the involved tooth. Periapical radiographic evaluation was done for the same, and a diagnosis of developmental dental anomaly of talon's cusp (type 1) or eagle's cusp was made as per Hattab and Yassin [10], along with the periapical abscess with respect to 61. The treatment plan was made as per the patient's comfort due to the young age. A pulpectomy of 61 followed by definite restoration using a strip crown was done. The patient was under regular follow-up (Figure 5).

## Discussion

Tooth development is a meticulously regulated and intricate biological process that relies on multiple factors for successful completion. Any disruptions during this process can lead to noticeable clinical changes or alterations (Table 1) [11]. Developmental dental abnormalities in primary teeth can cause a variety of distinct problems that alter their appearance and function. Despite multiple cases documented in the literature, distinguishing between these anomalies remains difficult. A proper case history, along with a clinical and radiographic examination, offers the necessary information for diagnosing such anomalies.

**Table 1:** Histopathological causes of developmental dental anomaly

Case	Anomaly	Histopathological disturbances
Case 1	Gemination and supernumerary tooth	Morpho-differentiation anomaly
Case 2	Fusion	Morpho-differentiation anomaly
Case 3	Gemination	Morpho-differentiation anomaly
Case 4	Taurodontism	Morpho-anatomical anomaly
Case 5	Talon's cusp	Morpho-differentiation anomaly

Double teeth describe both germination and fusion. These anomalies are more common in the primary dentition than in the permanent dentition. Fusion can impair tooth eruption, alignment, and spacing within the dental arch, potentially necessitating orthodontic treatment. A geminated tooth has a single pulp chamber and typically a single root canal, whereas fused teeth have a distinct pulp chamber with two roots or two root canals in a single root. Gemination typically results in crowding, whereas fusion causes ectopic eruption [13].

Supernumerary teeth are a rare finding in the primary dentition [1]. Supernumerary teeth can cause the delayed or impaired eruption of succedaneous teeth (26-52%), the displacement or rotation of permanent teeth (28-63%), crowding, abnormal diastema or premature space closure, dilaceration or the abnormal root development of permanent teeth, cyst formation (4-9%), or eruption into the nasal cavity [14,15]. Case 1 in the present series had a supernumerary tooth that resembled a lateral incisor. The patient is being followed up on a regular basis for self-exfoliation to ensure that no orthodontic problems arise in the near future with the permanent dentition.

According to Shifman and Chanannel, taurodontism can be diagnosed with the Taurodontic Index (TI), divided into three different degrees: hypotaurodontism, mesotaurodontism, and hypertaurodontism [12]. Taurodontism is distinguished by the elongation of the tooth body and enlargement of the pulp chamber, which frequently resemble the shape of a bull's molar. Endodontic procedures on taurodont teeth have been described as complex and demanding. Pulp therapy for taurodont teeth is a difficult procedure with an increased risk of bleeding during access opening, which might be misconstrued for perforation [16]. The case in the current case series had similar issues like an increased amount of pulpal tissue and tortuous canals which made the root canal procedure difficult. 

Talon cusps appear as an additional cusp-like structure extending from the cingulum area or the palatal surface of the anterior teeth, resembling an eagle's talon. Hattab and Yassin [10] divided talon cusps into three types, according to the extent of projection from the cemento-enamel junction (CEJ) toward the incisal edge, with a grading system of 1-3. In the present case series, a type 1 talon cusp was present (Figure 5). Early identification and therapy of talon's cusp are vital to prevent occlusal interference, caries of the developing grooves, impaired aesthetics, periodontal problems caused by high occlusal stresses, and irritation of the tongue during speaking and mastication [17]. The discovery of the talon cusp in this case was incidental, but later, the tooth underwent treatment for periapical pathology. When preparing the tooth for a strip crown, the talon cusp was reduced to ensure a smooth palatal surface.

Complications of anomalous primary teeth include an unaesthetic appearance of the affected teeth, greater susceptibility to caries, and malocclusion [18]. More importantly, defects in the primary dentition cause anomalies in the permanent teeth. Timely intervention would reduce difficulties in permanent dentition. The clinical implications of these anomalies vary, necessitating careful assessment and management to guarantee adequate dental health and function for the impacted primary teeth [19].

Clinical importance, strength, and limitation

Primary teeth serve as guides for the eruption of permanent teeth. Developmental anomalies in primary teeth can disrupt this process, leading to the malalignment or impaction of permanent teeth. It can lead to tooth structure weakness, making primary teeth more prone to caries, infection, and abscess formation. Anomalies affecting tooth shape, size, or number can affect its function and also impair phonetic articulation in children, leading to speech difficulties. The strength of this case series lies in its contribution to the existing literature by enhancing the understanding, diagnosis, and management of developmental anomalies that occur without associated syndromes and are therefore often overlooked in children. Early management will give better outcomes for future dentition. The limitation of this paper is the small number of cases currently being described due to the word limit of the journals, but the author encourages further reporting of such cases so as to create a substantial database for non-syndromic developmental anomalies in primary dentition. 

## Conclusions

Developmental dental anomalies in primary dentition, such as fusion, gemination, taurodontism, and talon's cusp, represent unique challenges in pediatric dental care. These anomalies, though relatively uncommon, can have significant implications for oral health, influencing both aesthetics and function. Early diagnosis through clinical and radiographic examination is crucial for effective management, allowing for timely interventions that can mitigate potential complications. Understanding the etiology and presentation of these anomalies enables dental professionals to formulate better treatment plans that address the specific needs of each child, promoting optimal oral health outcomes.
